# Clival Ectopic Pituitary Adenoma Mimicking a Chordoma: Case Report and Review of the Literature

**DOI:** 10.1155/2016/8371697

**Published:** 2016-01-13

**Authors:** Constantine L. Karras, Isaac Josh Abecassis, Zachary A. Abecassis, Joseph G. Adel, Esther N. Bit-Ivan, Rakesh K. Chandra, Bernard R. Bendok

**Affiliations:** ^1^The Ohio State University College of Medicine, Columbus, OH 43212, USA; ^2^Department of Neurological Surgery, University of Washington, Seattle, WA 98122, USA; ^3^Feinberg School of Medicine, Northwestern University, Chicago, IL 60611, USA; ^4^Department of Neurological Surgery, Northwestern University, Chicago, IL, USA; ^5^Department of Pathology, Feinberg School of Medicine, Northwestern University, Chicago, IL 60611, USA; ^6^Department of Otolaryngology, Feinberg School of Medicine, Northwestern University, Chicago, IL 60611, USA

## Abstract

*Background*. Purely ectopic pituitary adenomas are exceedingly rare. Here we report on a patient that presented with an incidental clival mass thought to be a chordoma. Endonasal resection, tumor pathology, and endocrinology workup revealed a prolactinoma.* Case Presentation*. A 41-year-old male presented with an incidental clival lesion presumed to be a chordoma. On MRI it involved the entire clivus, extended laterally to the petroclival junction, and invaded the cavernous sinuses bilaterally, encasing both internal carotid arteries, without direct extension into the sella. Intraoperatively, it was clear that the tumor originated from the clivus and that the sellar dura was completely intact. Frozen-section pathology was consistent with a pituitary adenoma. Immunostaining was positive for synaptophysin and prolactin with a low Ki-67 index, suggestive of a prolactinoma. Additional immunohistochemical stains seen in chordomas (EMA, S100, and Brachyury) and other metastatic tumors were negative. A postoperative endocrine workup revealed an elevated serum prolactin of 881.3 ng/mL (normal < 20).* Conclusions*. In conclusion, it is crucial to maintain an extensive differential diagnosis when evaluating a patient with a clival lesion. Ectopic clival pituitary adenomas, although rare, may warrant an endocrinological workup preoperatively as the majority may respond to medical treatment.

## 1. Introduction

Pituitary adenomas are the most common cause of a sellar or parasellar mass, comprising about 10 to 15 percent of all intracranial tumors [1, 2]. It is well documented that pituitary adenomas have the capacity to invade adjacent and nearby structures, most commonly the sphenoid sinus [3]. Less commonly, pituitary adenomas may also involve the cavernous sinus, suprasellar region, nasopharynx, and rarely the clivus [4]. Purely ectopic pituitary adenomas are extremely unusual and must be confirmed via imaging and intraoperative visualization to be completely separate from an intact pituitary gland and the sella turcica. Here we report on a patient that presented with an incidental clival mass thought to be a chordoma. Endonasal resection, pathology of the tumor, and endocrinology workup revealed a purely ectopic prolactin-secreting pituitary adenoma.

## 2. Case Report

Our patient was a 41-year-old right-handed male who presented with an incidental clival lesion, originally presumed to be a chordoma. Subjectively, the patient reported no neurological complaints. The patient's medical history was significant only for chronic neck and lower back pain and there were no noteworthy findings on physical examination, which included a thorough neurological exam. His complete blood count, chemistry panel, and metabolic panel were all normal.

**Figure 1:**
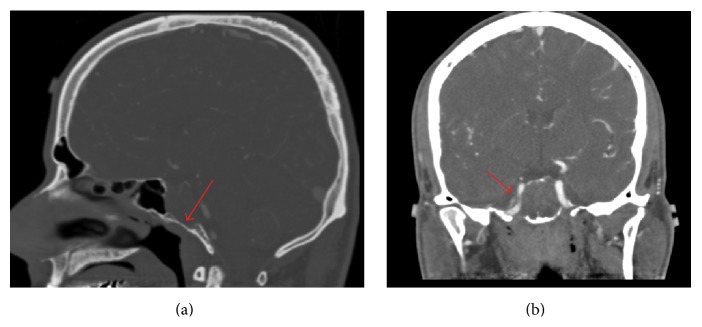
Preoperative computed tomography angiogram (CTA) scan, sagittal bone window (a) and coronal vascular window (b) sections, showing poorly vascularized clival mass that erodes the clivus (a) and encases the right internal carotid artery (b).

**Figure 2:**
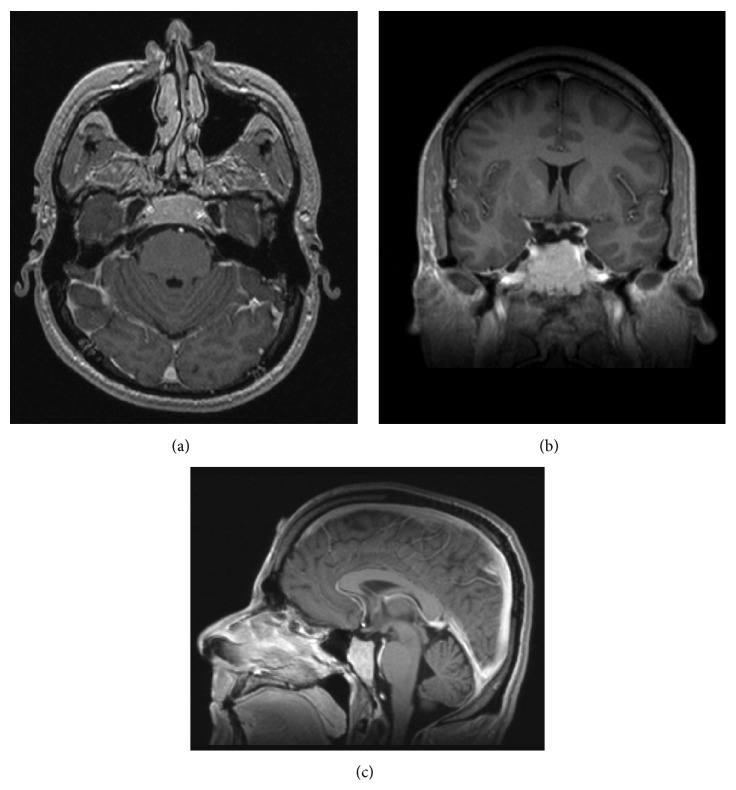
Preoperative magnetic resonance imaging, with contrast, axial (a), coronal (b), and sagittal (c) sections, demonstrating contrast enhancing, clival tumor.

Repeat magnetic resonance imaging (MRI) revealed an enhancing lesion measuring 1.7 × 2.6 × 3.2 cm that involved the entire clivus, extended laterally to the petroclival junction, and invaded the cavernous sinuses bilaterally with further encasement of the internal carotid arteries (ICAs) bilaterally, without direct extension into the sella (Figures 1(a)-1(b) and 2(a)–2(c)). This lesion was stable in comparison to outside hospital images. The case was discussed at an interdisciplinary brain tumor meeting and, ultimately, the patient was advised to undergo surgical resection via an endoscopic endonasal approach. Intraoperatively, it was very clear that the tumor originated from the clivus and that the sellar dura was completely intact. The frozen-section pathology surprisingly was consistent with a pituitary adenoma. Accordingly, a decision was made perioperatively to proceed with only partial resection due to the potential for medical management of most functional adenomas postoperatively and the risks associated with complete resection given the complex distribution of the lesion. The patient had no complications and was discharged home on postoperative day 2.

**Figure 3:**
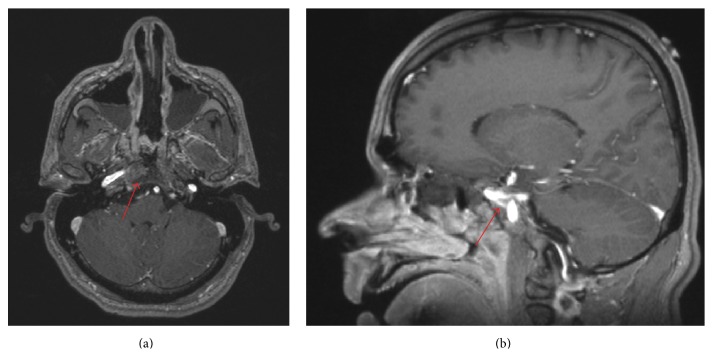
Postoperative magnetic resonance imaging, with contrast, axial (a) and sagittal (b) sections, demonstrating a small amount of residual tumor.

**Figure 4:**
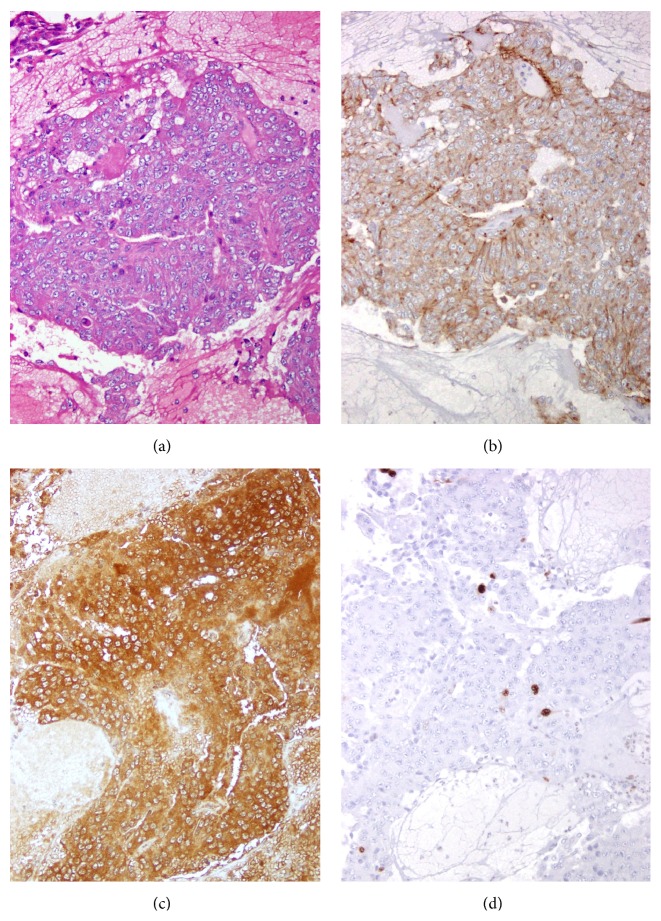
Pathologic evaluation, all at 20x magnification. (a) Histologic sections demonstrate an epithelial neoplasm composed of monomorphic cells with increased nuclear to cytoplasmic ratios and prominent nucleoli. (b) Immunohistochemical staining positive for synaptophysin. (c) Prolactin diffusely and strongly labels the neoplastic cells. (d) The proliferation index is approximately 2% on Ki-67 staining.

Postoperative imaging revealed expected residual enhancing lesions along the petroclival junctions bilaterally and on the right cavernous sinus near the right ICA (Figures 3(a) and 3(b)). Immunostaining of the tumor tissue demonstrated an epithelial neoplasm that stained positive for synaptophysin and prolactin with a low Ki-67 index (Figures 4(a)–4(d)), suggestive of a prolactin-secreting pituitary adenoma. Additional immunohistochemical stains seen in chordomas (EMA, S100, and Brachyury) and other metastatic tumors were negative. A postoperative endocrine workup (Table 1) revealed an elevated serum prolactin of 881.3 ng/mL (normal male < 20 ng/mL). The patient was referred to an endocrinologist and began dopamine agonist (cabergoline) therapy given his prolactin level and residual tumor. He has since been followed up for over 1 year with monthly visits. His most recent prolactin level (11 months postoperatively) was 20.6 ng/mL; his lowest level was 15.2 ng/mL, collected 6 months postoperatively. He remains neurologically intact.

## 3. Discussion

Clival tumors are generally rare, comprising 1% of all intracranial neoplasms. The differential diagnosis for a clival lesion is vast, including chordoma most commonly (40%), meningioma, chondrosarcoma, astrocytoma, craniopharyngioma, germ cell tumor, non-Hodgkin's lymphoma, melanoma, metastatic carcinoma, and rarely pituitary adenoma [4]. Our patient was presumed to have a chordoma based on the radiographic characteristics and the tumor location. Clival chordomas are typically managed with gross total resection with proton beam therapy. There has been one previous report of an ectopic pituitary adenoma thought to be a chordoma [12] and vice versa [4].

Ectopic pituitary adenomas are thought to arise from residual cells along the migration tract of the pharyngeal pituitary as it travels from Rathke's pouch to the sella turcica. The anterior portion of the pituitary initially develops as a pharyngeal outpouching of epithelial ectoderm, known as Rathke's pouch. Around week 8 of gestation, a portion separates and migrates through the craniopharyngeal canal into the sella, forming the anterior portion of the pituitary, from which most pituitary adenomas arise [9, 16]. Ectopically deposited cells can rarely develop into adenomas anywhere along this tract, though. There have been over 100 descriptions of ectopic pituitary adenomas in the literature, most originating in the sphenoid sinus [17]. The first reported, in 1901, was an ectopic pituitary adenoma [10] located in the sphenoid sinus that presented with acromegaly. However, purely ectopic clival pituitary adenomas—that is, tumors that originate in the clivus with no involvement of the pituitary gland—are exceedingly rare.

We encountered 16 prior reported cases of clival ectopic pituitary adenomas (Table 2) [4–9, 11–14, 17–21]. They seem to be fairly equally distributed between genders (9 males, 8 females) and the median age at presentation was 50 years old.

Like pituitary adenomas, ectopic tissue can be categorized by size as either a macroadenoma (>1 cm) or microadenoma (<1 cm). The tumor can be further classified as functional (65% of nonectopic adenomas) or nonfunctional based on whether or not the cell type is hormone-secreting. Prolactin-secreting pituitary adenomas (nonectopic) are the most common and comprise 48% of all functional adenomas [22]. Occasionally, pituitary adenomas can lack the cardinal features found on light microscopy and immunohistochemical staining that were present in our case. Electron microscopy of these tumors may aid in further classification of the cell type based on appearance of the nucleus, cytoplasm, organelles (including rough endoplasmic reticulum and Golgi apparatus), and granule morphology [7].

Analysis of Table 2 reveals that 10/17 clival ectopic pituitary tumors described were prolactinomas, 2/17 secreted ACTH, 2/17 secreted GH, and only 4/17 were nonfunctional—one tumor secreted both prolactin and GH [8]. Some (especially nonfunctional tumors) presented asymptomatically or with focal neurological deficits (5/17 cases) due to compression of nearby structures. The most common theme among patient presentations in the literature review was “frequent headache” in the period leading up to diagnosis. Pituitary adenomas often classically present with bitemporal hemianopsia, but ectopic adenomas typically do not unless they happen to involve the optic chiasm. One case presented as pituitary apoplexy—tumor infarction or hemorrhage resulting in a constellation of symptoms that may include headache, visual deficits, ophthalmoplegia, or altered mental status [13]. Most of these findings are nonspecific, however, and cannot distinguish a pituitary adenoma from other lesions such as a chordoma.

Fortunately, a surprising 76% (13/17) of reported cases were functional adenomas, permitting a possible preoperative diagnosis based on history, physical exam, and basic labs alone. ACTH-secreting adenomas may present with Cushing's syndrome (moon facies, truncal obesity, abdominal striae, hirsutism, etc.), characterized by hypercortisolism and an abnormal dexamethasone suppression test. GH-secreting adenomas may manifest as acromegaly in adults, which presents with enlargement of the extremities, coarse facies (frontal bossing, prognathism), carpal tunnel syndrome, diabetes, or cardiomyopathy—labs would reveal elevated IGF-1 (insulin-like growth factor) and a failed oral glucose tolerance test. Prolactinomas, which comprised the majority of cases (10/17), may present with gynecomastia, erectile dysfunction, decreased libido, galactorrhea, amenorrhea (in females), and an elevated prolactin level. Of note, after a more focused history following our postoperative diagnosis of prolactinoma, it was discovered retrospectively that our patient had actually had a significant drop in libido throughout the previous 7-8 years.

Our case was unique in that it was the first reported clival ectopic pituitary adenoma that invaded both cavernous sinuses and the internal carotid arteries, making it the most aggressive tumor of all reported cases—this is unusual for adenomas, as they typically are quite less infiltrative. This is why it was originally confused for a chordoma, but it is particularly important to maintain pituitary adenomas on the differential for sphenoidal or clival lesions because the diagnosis can have significant implications on the management of the tumor. For example, dopamine agonists (cabergoline, bromocriptine) and somatostatin analogs (octreotide, lanreotide) or GH antagonists (pegvisomant) are first-line therapy for prolactinomas and GH-secreting adenomas, respectively, depending on the clinical symptoms and anatomical distribution of the tumor. They may be used as monotherapy or as adjunctive treatment for tumor shrinkage. With 76% (13/17) of reported clival ectopic adenomas being hormone-secreting and 85% (11/13) of these being susceptible to medical management (GH- or prolactin-secreting), a significant opportunity exists for more conservative management if the diagnosis can be made preoperatively rather than retrospectively based on histology. This is especially useful for more aggressive tumors (such as our patient's, which invaded the cavernous sinus), where a complete resection may carry significant risk of bleeding and morbidity. Adjunctive pharmacotherapy can also potentially delay or even eliminate the need for surgery, especially in elderly individuals or in those with significant medical comorbidities and relative contraindications to surgery.

A common theme among reported cases, including ours, was that the correct diagnosis was made histologically rather than preoperatively. Management could have been drastically altered in most of these cases had a more focused, thorough history and physical exam alone occurred.

## 4. Conclusions

In conclusion, it is important to maintain an extensive differential diagnosis when evaluating a patient with a clival lesion. Ectopic clival pituitary adenomas, although rare, warrant consideration as the majority respond to pharmacotherapy [14] and thus are managed much differently than lesions such as chordomas. The diagnosis can easily be screened for with a focused history and physical exam directed toward symptoms of the most common types (prolactin-, GH-, and ACTH-secreting adenomas), which make up the majority (76%) of reported clival ectopic adenomas. If the history and physical exam yield a positive screen, a preoperative endocrinological workup is necessary to confirm the diagnosis.

## Figures and Tables

**Table 1:** Patient's endocrinological lab values.

	Patient	Reference range
Free T4 (ng/dL)	0.7	0.7–1.5
TSH (*μ*IU/mL)	2.58	0.4–4.0
Prolactin (ng/mL)	881.3	2.6–13.1
FSH (mlU/mL)	3	1.0–8.0 (Men)
LH (mlU/mL)	3.1	2.0–12.0 (Men)
Cortisol, 2PM (*μ*g/dL)	6.8	0–25
Total testosterone (ng/dL)	290	250–1100
Free testosterone (pg/mL)	58.4	35–155

T4 = thyroxine hormone.

TSH = thyroid stimulating hormone.

FSH = follicle stimulating hormone.

LH = luteinizing hormone.

**(a):** 

Authors	Patient age and gender	Immunostaining	Initial presentation
Ortiz-Suarez and Erickson, 1975 [11]	15 F	ACTH	Obesity, irregular menstrual cycles, increased facial hair, episodic headaches, and facial numbness
Shenker et al., 1986 [21]	49 M	Prolactin	Worsening renal failure, hypercalcemia, duodenal ulcer, parathyroid hyperplasia, fatigue, muscle pains, vomiting, and impotence
Anand et al., 1993 [18]	58 F	ACTH	Nasal obstruction, blurred vision, anosmia, and headache
Mount et al., 1993 [20]	71 M	Prolactin	Aphasia and R hemiplegia
Arnesen and Scheithauer, 1994 [7]	40 M	Prolactin	Bloody, mucoid nasal discharge, and nasal obstruction
Kikuchi et al., 1994 [12]	49 F	Null cell	Headaches, nausea, and vomiting after neck injury; incidentally discovered
Wong et al., 1995 [4]	67 M	Null cell	Unknown
De Witte et al., 1998 [19]	47 F	Prolactin	Headache
Hori et al., 1999 [17]	63 M	Null cell	Visual disturbances
Ballaux et al., 1999 [14]	80 F	Prolactin	Minor headache and transient amnesia
Sakakibara et al., 2002 [6]	70 F	Prolactin	Progressive L sided exophthalmos
Bhatoe et al., 2007 [5]	35 F	GH	Dull generalized headache, acral enlargement, weight gain, and coarsening facial features
Rocque et al., 2009 [9]	20 M	Prolactin	Bilateral gynecomastia and galactorrhea
Appel et al., 2012 [8]	50 F	GH/prolactin	Daily headaches, impaired concentration, fatigue, generalized muscle/joint pain, and acromegalic facial features
Mudd et al., 2012 [14]	78 M	Null cell	Acute onset blurred vision, apoplexy
Narese et al., 2015 [4]	65 M	Prolactin	Right ptosis, eyelid edema, and headache
This paper	41 M	Prolactin	Incidental imaging due to chronic neck and lower back pain

**(b):** 

Focal neurological findings	Imaging	Abnormal preoperative labs (ng/mL)	Treatment	Follow-up; outcomes
Oculomotor and trigeminal nerve palsies	Skull XR: mottling, sclerosis of sella turcica, and lesser wing of sphenoid. CT: normal. Carotid angiogram: medial displacement of ICA	None	Right transfrontal craniotomy + 5000 Rads	1 year; returned to baseline

None	Skull XR: enlarged sella, CT: partially empty sella, destroyed sella floor, and mass at base of sella with invasion into sphenoid sinus	PRL = 1900	Endonasal transsphenoidal resection + cabergoline	1 year; no recurrence, impotence resolved

L eye inferior medial quadrant visual field defect	MRI: 3 × 3 cm, midline homogenous mass filling posterior nasopharynx and clivus	None	Total resection via open-door maxillotomy approach + 4550 Rads over 25	1 year; complete resolution of symptoms

Unknown	CT/MRI: L frontotemporal hematoma, meningioma, expansile density with invasion into sphenoid bone and clivus, and encasing ICAs	None	Endonasal transsphenoidal biopsy only + radiation	No improvement, transferred to receive supportive care

Unknown	MRI: tumor eroding through skull base into the clivus extending into sphenoid sinus, cavernous sinus, and surrounding ICA	Unknown	Partial endonasal transsphenoidal resection	Unknown

None	Skull XR: normal size sella, slight erosion of floor CT/MRI: large enhancing mass in sphenoid sinus invading sphenoid wing and clivus	None	Partial resection via sublabial transnasal approach + 50 Gy radiation/6 wks	Unclear; “under careful observation”

Unknown	MRI: clival destruction	PRL = 7	Unknown	Unknown

None	CT/MRI: clival lesion, destruction of bone	None (Post-op PRL = 34,000)	Endonasal transsphenoidal partial resection + bromocriptine	4 months; normalization of lab values

Bitemporal hemianopsia	CT: lesion in extradural sella-clivus region	Unknown	Transfacial surgery	Unknown

None	CT: tumor at clivus with surrounding bony destruction. MRI: enhancing mass with cystic component, invading sphenoid sinus	PRL = 2519.8	Cabergoline only	6 months; resolution of lab values and symptoms

Exophthalmos with external ocular movement disorders and decreased visual acuity on L	CT: bony destruction of clivus, sphenoid sinus, and medial aspect of middle cranial fossa, MRI: abnormal enhancement in sphenoid sinus	PRL = 645.7	Endonasal transsphenoidal resection + Bromocriptine therapy	1 year f/u; resolution of visual symptoms

Unknown	Skull radiograph: normal, MRI: clival mass connected to intrasellar lesion	GH = 30.6	Endonasal transsphenoidal resection	1 year; normalization of lab values

None	MRI: 13 mm erosive mass in clivus with focal area of bony erosion	PRL = 178	Endonasal transsphenoidal total resection	6 months; complete resolution of symptoms

None	MRI: 2 mm hypointense lesion on pituitary gland. Clival lesion discovered incidentally during surgery	IGF-1 = 937, PRL = 26	Endoscopic transsphenoidal; clival mass encountered and resected, pituitary unremarkable	3 months; normalization of lab values, no report on clinical status

L CN 6 palsy	MRI: lytic lesion of left clivus, compression of cavernous sinuses, clival mass, and normal sella	None	Endoscopic transsphenoidal resection	2.5 years; resolution of CN6 palsy and no recurrence

None	MRI: large tumor at height of clivus, partial destruction of surrounding bone structure	Unknown	Endoscopic transsphenoidal resection	Unknown

None	MRI: enhancing lesion in clivus with extension into cavernous sinuses and encasement of the ICAs	PRL = 881.3	Endoscopic transsphenoidal; subtotal resection and dopamine antagonist	1 year; no symptoms

F = female.

M = male.

GH = growth hormone.

PRL = prolactin.

ACTH = adrenocorticotropic hormone.

IGF-1 = insulin-like growth factor-1.

MRI = magnetic resonance imaging.

CT = computed tomography.

XR = X-ray.

ICAs = internal carotid arteries.

L = left.

CN = cranial nerve.
